# Hippocampal 5-HT_1A_ Receptor and Spatial Learning and Memory

**DOI:** 10.3389/fphar.2015.00289

**Published:** 2015-12-10

**Authors:** Yifat Glikmann-Johnston, Michael M. Saling, David C. Reutens, Julie C. Stout

**Affiliations:** ^1^Faculty of Medicine, Nursing and Health Sciences, School of Psychological Sciences, Monash UniversityMelbourne, VIC, Australia; ^2^Department of Neuropsychology, Austin HealthMelbourne, VIC, Australia; ^3^Faculty of Medicine, Dentistry and Health Sciences, Melbourne School of Psychological Sciences, The University of MelbourneMelbourne, VIC, Australia; ^4^Centre for Advanced Imaging, The University of QueenslandBrisbane, QLD, Australia

**Keywords:** serotonin, 5-HT_1A_ receptor, hippocampus, spatial cognition, memory

## Abstract

Spatial cognition is fundamental for survival in the topographically complex environments inhabited by humans and other animals. The hippocampus, which has a central role in spatial cognition, is characterized by high concentration of serotonin (5-hydroxytryptamine; 5-HT) receptor binding sites, particularly of the 1A receptor (5-HT_1A_) subtype. This review highlights converging evidence for the role of hippocampal 5-HT_1A_ receptors in spatial learning and memory. We consider studies showing that activation or blockade of the 5-HT_1A_ receptors using agonists or antagonists, respectively, lead to changes in spatial learning and memory. For example, pharmacological manipulation to induce 5-HT release, or to block 5-HT uptake, have indicated that increased extracellular 5-HT concentrations maintain or improve memory performance. In contrast, reduced levels of 5-HT have been shown to impair spatial memory. Furthermore, the lack of 5-HT_1A_ receptor subtype in single gene knockout mice is specifically associated with spatial memory impairments. These findings, along with evidence from recent cognitive imaging studies using positron emission tomography (PET) with 5-HT_1A_ receptor ligands, and studies of individual genetic variance in 5-HT_1A_ receptor availability, strongly suggests that 5-HT, mediated by the 5-HT_1A_ receptor subtype, plays a key role in spatial learning and memory.

## Introduction

The idea that serotonin (5-hydroxytryptamine; 5-HT) is involved in learning and memory has gained traction in recent years, after having first been suggested in the 1980s (Altman and Normile, 1988). Early pharmacological studies mostly implicated spatial memory. More recent studies involving advanced methodologies such as neurotransmitter positron emission tomography (PET) and knockout mouse models have continued to link serotonin to spatial memory.

Spatial memory includes the ability to learn the topographical configuration of environments, to locate objects, to recall previously encountered locations, and to navigate within environments. Many day-to-day activities performed by animals and humans depend on spatial memory. Knowing where one is, where food and water resources are, and how to get to safety are examples of effective use of spatial memories that are essential for animal survival. Humans depend on their ability to remember the locations of objects in the environment on a daily basis, ranging from retrieving a mobile phone from a purse to making one's way to work and back home (McNamara, 2013).

At a clinical level, the study of spatial memory is of particular significance to several neurological disorders such as dementia of the Alzheimer's type where impairments in spatial cognition are a central feature. In addition, spatial memory, and particularly the ability to process and remember spatial descriptions of environments, has been linked to certain types of learning disabilities in children (Mammarella et al., 2014).

Functional neuroimaging studies show that spatial memory is largely mediated by mesial temporal areas (for example, Maguire et al., 1996b, 1997, 1998a,b; Burgess et al., 2001; Hartley et al., 2003), and within these areas, the hippocampus is a key structure for spatial memory. These regions are characterized by high concentration of the 5-HT_1A_ receptor binding sites.

Involvement of the 5-HT_1A_ receptor in cognition is undisputed. This receptor subtype has been suggested as a therapeutic target and neural marker of memory deficits (Meneses, 1999; Meneses and Perez-Garcia, 2007; Thomas, 2015). In this review, we argue that the 5-HT_1A_ receptor plays a key role in spatial learning and memory, and we present evidence to support this proposition. We first consider the correspondence between the neuroanatomy of spatial memory and the 5-HT_1A_ receptor distribution. We then review studies using various experimental methods that have illustrated the role of 5-HT_1A_ receptors in spatial learning and memory.

## Neuroanatomy of spatial learning and memory

Research on spatial memory has consistently implicated a hippocampal brain network consisting of the hippocampus proper, the parahippocampal cortices, fornix, parietal cortex, anterior thalamic nuclei, frontal cortex, and the striatum. The critical role of the hippocampal system in spatial learning and memory was first highlighted by Brenda Milner's early observations of “heightened” spatial memory deficits following temporal lobe excision for the relief of epileptic seizures (Milner, 1958, p. 251). Evidence for the importance of the hippocampus system has continued to accumulate, including very recent findings using single-neuron recording in human entorhinal cortex during virtual navigation (Miller et al., 2015). In terms of possible brain mechanisms underlying spatial learning and memory, findings have indicated that the rat hippocampus contains “place cells,” and these cells exhibit location-specific activity (O'Keefe and Dostrovsky, 1971; O'Keefe and Speakman, 1987). This discovery led to the hypothesis that the hippocampus stores a cognitive map of the spatial layout of the environment (O'Keefe and Nadel, 1978). More than three decades later, in 2005, “grid cells” were found in the rat's entorhinal cortex, which is the chief gateway into the hippocampus (Hafting et al., 2005). Grid cells generate a coordinate system that allows exact positioning and pathfinding. Together with other cells in the entorhinal cortex that recognize the direction of the head of the animal and the border of the environment (“head-direction cells”; Taube, 1998), grid cells form networks with place cells in the hippocampus. Overall this circuitry constitutes a comprehensive positioning system, an inner global positioning system, or GPS, in the brain.

In addition to these cell recording studies, lesions and stimulation of the hippocampus in non-human primate (Parkinson et al., 1988; Angeli et al., 1993) and rodents (Morris et al., 1982; Buhot et al., 1991) were shown to impair spatial learning and memory. Similarly, in humans, medial temporal lesions, especially on the right side, have been shown to impair recall of spatial location of objects (Smith and Milner, 1981, 1989; Pigott and Milner, 1993; Bohbot et al., 1998; Smith et al., 2011), increase spatial memory errors (using the None-Box Maze, Abrahams et al., 1997, 1999), and impair performances on virtual reality topographical memory tasks (Spiers et al., 2001b).

More precise links between particular spatial memory functions and regions within the hippocampal network have been established in some studies. For example, early studies indicated lateralization of hippocampal involvement in memory, with the right medial temporal lobe predominantly associated with visuospatial recall (for example, Milner, 1965; Smith and Milner, 1981, 1989; Pigott and Milner, 1993; Abrahams et al., 1997; Maguire et al., 1997; Gleissner et al., 1998; Lv et al., 2014), and the left medial temporal lobe with verbal material recall (for example, Saling et al., 1993; Hermann et al., 1997; Martin et al., 2002; Lillywhite et al., 2007). In keeping with this idea, a patient with Pick's disease involving the left temporal lobe showed a complete dissociation between topographical memory and verbal memory (Maguire and Cipolotti, 1998), although more recent findings (for example, Maguire et al., 1996a,b; Grön et al., 2000; Spiers et al., 2001a; Astur et al., 2002; Glikmann-Johnston et al., 2008; Cánovas et al., 2011) support involvement of both the left and right medial temporal lobes in spatial learning and memory.

The cortices adjacent to the hippocampus, which provide the hippocampus with its main source of direct cortical input and output, have also been implicated in spatial learning and memory. For example, some studies indicated bilateral involvement of the parahippocampal gyri (Aguirre et al., 1996, 1998; Aguirre and D'Esposito, 1997; Epstein and Kanwisher, 1998; Mellet et al., 2000; Zeidman et al., 2012), whereas other studies indicate unilateral, predominantly right-sided involvement (Habib and Sirigu, 1987; Owen et al., 1996; Bohbot et al., 2000; Ploner et al., 2000). In terms of other regions of the hippocampal formation, in non-human primates, cells in the entorhinal cortex are active during the performance of a variation of the delayed matching to sample task (memory for objects) and the delayed matching to place task (memory for place) (Suzuki et al., 1997). Location-specific activity of neurons has also been recorded within the rat entorhinal cortex (Quirk et al., 1992). Furthermore, lesions to the entorhinal cortex in rats have been shown to result in deficits in acquisition and retention of the Eight-Arm Radial Maze and the Morris Water Maze (Cho and Jaffard, 1995; Nagahara et al., 1995; Davis et al., 2001; Devi et al., 2003). In humans, entorhinal stimulation applied during learning the locations of landmarks enhanced subsequent memory for these locations (Suthana et al., 2012). In a single-neuron recording study, entorhinal cortex neurons activated at multiple related areas of a virtual environment (Miller et al., 2015). Combined lesions of entorhinal and perirhinal cortices impaired rats' performance in spatial memory tasks (Otto et al., 1997; Kaut and Bunsey, 2001). In contrast, perirhinal lesions alone yielded inconsistent results, with some studies showing impaired performance in certain tests of spatial memory (Wiig and Bilkey, 1994a,b; Liu and Bilkey, 1998a,b,c, 1999, 2001), while in others spatial memory was spared (Glenn and Mumby, 1998; Bussey et al., 1999, 2001; Machin et al., 2002; Ramos, 2002, 2013; Moran and Dalrymple-Alford, 2003). Thus, involvement of the perirhinal cortex in spatial learning and memory may be related to the specific memory paradigm employed.

**Figure 1:**
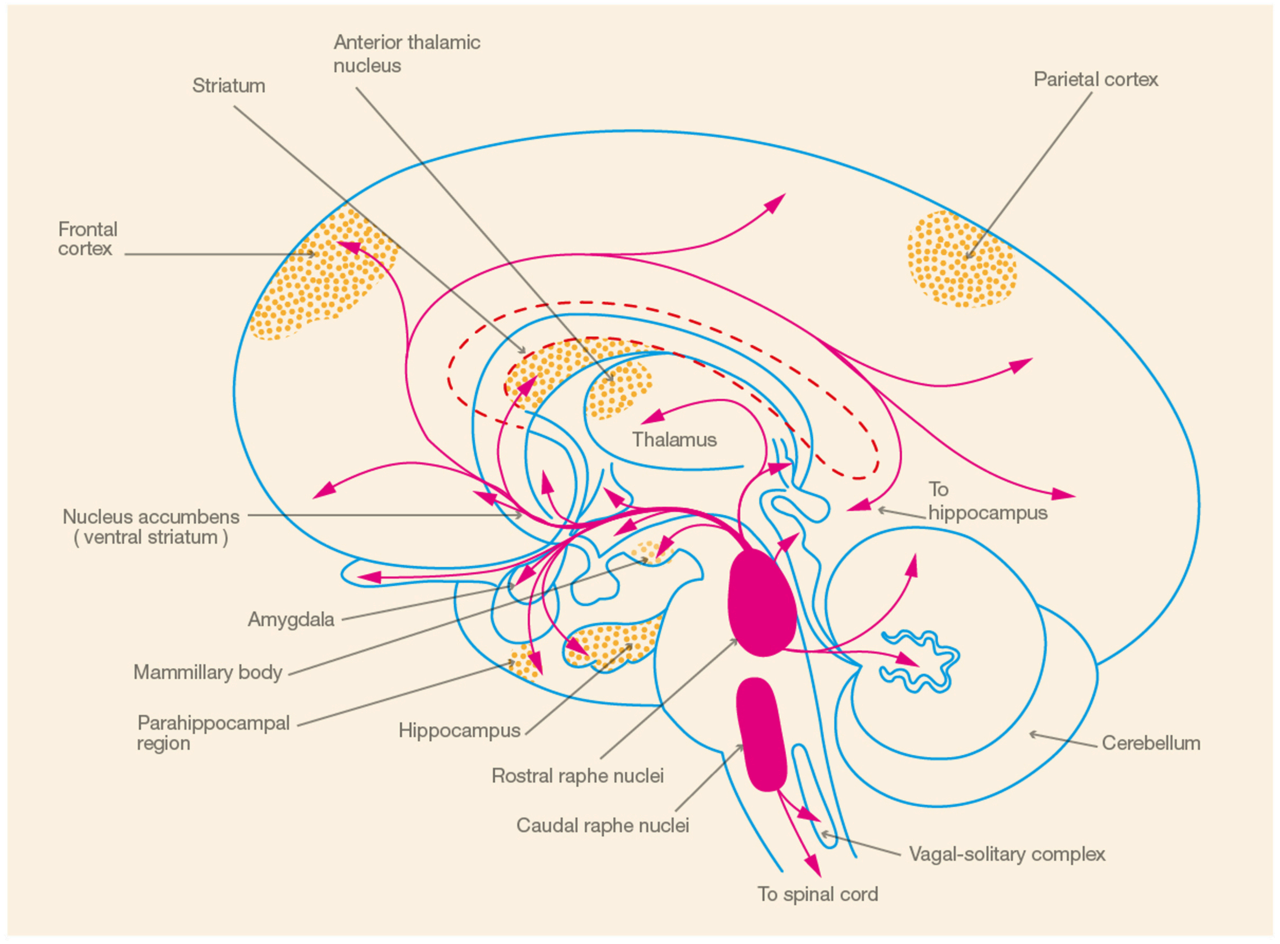
**Schematic illustration of brain areas involved in spatial memory (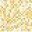) and the corresponding serotonergic pathways (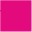) (Adapted from Heimer, 1994, p. 227)**.

In the following section, we provide an overview of 5-HT synthesis, electrophysiology, and receptor distribution to illustrate the concordance between 5-HT receptor distribution and brain areas involved in spatial memory, focusing on the hippocampus (see Figure 1). Subsequently, we review the evidence that 5-HT, mediated by the 5-HT_1A_ receptor, is involved in the modulation of spatial learning and memory.

## Serotonin (5-Hydroxytryptamine; 5-HT) and the 5-HT_1A_ receptor

Neurons that synthesize 5-HT are clustered in several nuclei along the midline of the brainstem, the most prominent of which are the raphe nuclei. Axons of these neurons innervate almost all regions of the central nervous system (CNS) and thus affect a great variety of behaviors, such as sleep/wake cycle, food intake, sexual behavior, emotional state, and cognitive processes, particularly learning and memory (Frazer and Hensler, 1994). 5-HT is synthesized from the amino acid tryptophan. The initial step in synthesis is the conversion of tryptophan to 5-hydroxytryptophan (5-HTP) by the enzyme tryptophan hydroxylase. Aromatic amino acid decarboxylase (AACD) then converts 5-HTP to 5-HT. 5-HT release occurs via exocytosis and is Ca^2+^-dependent. After 5-HT release, the actions of 5-HT in the synapse are terminated by 5-HT transporters, located on the plasma membrane of serotonergic neurons, which reuptake 5-HT back into the serotonergic neurons. 5-HT catabolism occurs by monamine oxidase A (MAO-A) (Frazer and Hensler, 1994; Adell et al., 2002).

Seven types of 5-HT receptors have been identified, termed 5-HT_1-7_, and among these are 14 distinct receptor subtypes. Each 5-HT receptor subtype has unique structural and pharmacological characteristics and a distinct distribution in the CNS. Of special interest is the 5-HT_1A_ receptor, which is highly concentrated within the hippocampal system. 5-HT_1A_ receptors are mainly concentrated in the limbic system, particularly the hippocampus (dentate gyrus and CA1), lateral septum, and amygdala, in cingulate and entorhinal cortices, and in the dorsal and median raphe nuclei, many of the regions implicated in spatial learning and memory. In contrast, only low concentrations are present in the striatum, substantia nigra, and the cerebellum (Barnes and Sharp, 1999; Lanfumey and Hamon, 2000). Autoradiography and immunohistochemical methods show that 5-HT_1A_ receptors are located post-synaptically, as well as on the serotonergic neurons themselves in the raphe nuclei where they act as somatodendritic autoreceptors (Verge et al., 1985, 1986; Hoyer et al., 1986; Pazos et al., 1987; Zifa and Fillon, 1992; Hall et al., 1997; Lanfumey and Hamon, 2000). At the cellular level, 5-HT_1A_ receptors reside on hippocampal pyramidal and granule cells (Lanfumey and Hamon, 2000). The highest density of these receptors are found in the granular layer (Hall et al., 1997).

In both hippocampus and dorsal raphe regions, 5-HT_1A_ receptor activation results in neuronal hyperpolarization through the interaction with G-protein and the opening of K^+^ channels (Hamon et al., 1990; Frazer and Hensler, 1994; Lanfumey and Hamon, 2000). Since 5-HT_1A_ receptors are located pre- and post-synaptically, endogenous 5-HT and/or 5-HT_1A_ receptor agonists have different effects. 5-HT_1A_ somatodentritic autoreceptors modulate synaptic transmission. When activated via endogenous 5-HT and/or 5-HT_1A_ receptor agonists, they inhibit the serotonergic neuron on which they reside, and reduce 5-HT release. In contrast, at post-synaptic receptors such as occur in the hippocampus, 5-HT_1A_ agonists facilitate 5-HT neurotransmission (Lanfumey and Hamon, 2000). Brain areas that are critical for spatial learning and memory, such as those that are part of the hippocampal formation, harbor the post-synaptic 5-HT_1A_ receptors.

## 5-HT_1A_ and spatial learning and memory

Evidence to support a role for the 5-HT_1A_ receptor in spatial learning and memory comes from a variety of experimental methods, including mouse “knockout” models, direct receptor activation and blockade, neurotransmitter PET imaging, genetic studies, and manipulation of 5-HT concentrations. We organize this review according to the primary experimental method used. Studies cited here are summarized in Table 1.

**Table 1:** **Summary of the studies cited according to the experimental method used**.

**Method**	**Citation**	**Findings**
Knockout mouse models	Sarnyai et al., 2000	5-HT_1A_-deficient mice were impaired on the Morris water maze and the “Y” shape maze.
	Wolff et al., 2004	Young-adults, but not aged, 5-HT_1A_ knockouts exhibited an impairment of learning and retention of the Morris water maze.
5-HT_1A_ receptor stimulation	Micheau and Van Marrewijk, 1999	Intra-peritoneal administration of 8-OH-DPAT^a^ improved acquisition of a spatial discrimination task in an 8-arm radial maze. Intra-septal administration produced the same pattern of findings, but the improvement was less pronounced.
	Bertrand et al., 2000	Intra-septal injection of 8-OH-DPAT significantly impaired spatial learning in a water maze task.
	Warburton et al., 1997	Effects of 8-OH-DPAT on the rat performance of the Delayed Non-Matching to Position task varied according to the site of administration. In the dorsal raphe, the compound had no effect at any dose. Administration into the median raphe improved performance accuracy. In the dorsal hippocampus, 8-OH-DPAT produced a small impairment in performance.
	Carli et al., 1995	Rats treated subcutaneously with 8-OH-DPAT were impaired in choice accuracy on a two-platform spatial discrimination task. Spiroxatrine and (+)WAY100135^b^ prevented the impairment of accuracy caused by 8-OH-DPAT.
	Egashira et al., 2006	Bilateral microinjections of 8-OH-DPAT into rats' dorsal hippocampus impaired spatial memory on the eight-arm radial maze. WAY-100135 and NAN-190^c^ reversed the spatial memory impairment produced by 8-OH-DPAT.
	Olsen et al., 2012	Chronic treatment with buspirone^d^ in rats attenuated traumatic brain injury-induced spatial learning and memory impairments on the Morris water maze.
	Cheng et al., 2008	Delayed and chronic treatment regimen with 8-OH-DPAT after cortical impact injury in rats facilitated motor recovery and acquisition of spatial learning in a water maze task.
	Monaco et al., 2014	A combined therapeutic regimen of buspirone and environmental enrichment was more effective than either alone in enhancing spatial learning in brain injured pediatric rats.
Imaging serotonergic neurotransmission	Glikmann-Johnston et al., 2015	Hippocampal asymmetry in PET [^18^F]MPPF binding was associated with performance on a virtual object-location task. A lower binding potential in the right vs. the left hippocampus was related to better memory performance.
	Theodore et al., 2012	Using the PET ligand 18FCWAY, reduced left hippocampal 5-HT_1A_ receptor binding in temporal lobe epilepsy patients was related to delayed auditory verbal memory impairment, independent of the side of the epileptic focus.
Genetic variance in 5-HT_1A_ receptor availability	Roiser et al., 2006	MDMA^e^ users and controls who are carriers of the *S* allele at the 5-HT transporter gene-linked polymorphic region (5-HTTLPR)^f^ outperformed the *L* allele carriers on a visuo-spatial planning task, independent of drug use.
	Roiser et al., 2007	Carriers of the *S* allele at the 5-HTTLPR were more accurate than carries of the *L* allele on the CANTAB Pattern Recognition Memory.
	Jedema et al., 2010	Rhesus monkeys who are *S* allele carriers of the 5-HTTLPR were more accurate than carriers of the *L* allele on the delayed match to sample task.
Manipulations of 5-HT levels	du Jardin et al., 2014	PCPA^g^ induced 5-HT depletion in rats and caused memory deficits on object recognition and Y-maze spontaneous alternation tests. Flesinoxan^h^ significantly occupied 5-HT_1A_ receptors and restored PCPA-induced spatial memory deficits.
	Fox et al., 2000	Heavy MDMA users were impaired on several spatial memory components as tested by the CANTAB, including pattern recognition and spatial working memory.
	Skelton et al., 2006	MDMA-treated rats showed long lasting spatial learning deficits on the Morris water maze. Their performance on the Cincinnati water maze, a test of path integration learning, was initially impaired, but recovered over time.
	Vorhees et al., 2007	MDMA dose distribution had a long-term differential effect on different types of spatial learning. Path integration was mostly impaired following administration of a single dose. Spatial learning and reference memory was significantly impaired following administration of four divided doses.
	Fisk et al., 2011	Current and previous MDMA use was associated with visuospatial working memory impairment.
	Morford et al., 2002	Neonatal rats treated with D-fenfluramine^i^ on the 11th–20th post-natal days exhibited infantile and adult spatial learning and memory deficits in the Morris water maze, and sequential learning impairments in a Cincinnati water maze.
	Vorhees et al., 1994	Rats exposed to methamphetamine^j^ in early or late post-natal development exhibited impaired performance on a complex T-maze and on the Morris water maze.
	Vorhees et al., 2000	Neonatal methamphetamine treatment in rats produced selective spatial learning and memory deficits on the Morris water maze during adulthood.
	Vorhees et al., 2008	Methamphetamine treatment in rats impaired path integration learning irrespective of dose. Only high doses of the drug impaired rats' spatial learning and memory performance on the Morris water maze. Enrichment of rearing conditions significantly improved acquisition of the task.
	Schröder et al., 2003	A neurotoxic regimen of methamphetamine rats induced damage to 5-HT terminals, as indicated by decreased [^125^I]RTI-55 binding in the hippocampus, and impaired performance on an object recognition task, but not performance in the Morris water maze.

a *8-OH-DPAT is a 5-HT_1A_ receptor agonist*.

b *Spiroxatrine and (+)WAY100135 are 5-HT_1A_ receptor antagonists*.

c *NAN-190 is a 5-HT_1A_ receptor antagonist*.

d *Buspirone is a 5-HT_1A_ receptor agonist*.

e *Acute effects of MDMA include a rapid and significant increase in 5-HT, released from presynaptic vesicular stores. Repeated and high doses of MDMA cause decreased concentrations of 5-HT and its metabolite 5-HIAA*.

f *The S allele at the 5-HTTLPR is associated with reduced serotonergic neurotransmission relative to the L allele*.

g *Parachlorophenylalanine (PCPA) inhibits tryptophan hydroxylase, and thus reduces 5-HT synthesis*.

h *Flesinoxan is a selective 5-HT_1A_ receptor agonist*.

i *D-fenfluramine is a substituted amphetamine that induces 5-HT release and inhibits its reuptake. Initially, D-fenfluramine increases 5-HT extracellular concentrations, but later causes a significant depletion*.

j *Methamphetamine induces long-lasting reductions of dopamine and 5-HT, inhibits presynaptic neurotransmitter reuptake, and reduces tyrosine and tryptophan hydroxylase activities*.

### Knockout mouse models

Studies using genetically modified animals, particularly those of single gene deletions in knockout mice, provide the strongest evidence for the role of the 5-HT_1A_ receptor in learning and memory (see Bert et al., 2008 for a review of learning and memory in 5-HT1A-receptor mutant mice). Sarnyai et al. (2000) assessed 5-HT_1A_-deficient mice on hippocampal-related spatial learning and memory tasks, the Morris Water Maze and the “Y” shape Maze. Their results showed that lack of 5-HT_1A_ receptors is specifically associated with spatial learning and memory impairments. Wolff et al. (2004) demonstrated similar impairments in learning and retention of the Morris Water Maze in young-adult 5-HT_1A_ knockout mice, but not in aged 5-HT_1A_ knockout mice. The authors suggested that the reduced effect of the mutation in aged animals possibly reflects the lower efficacy of autoreceptors due to aging and/or a prevalence of hippocampal heteroreceptors.

### 5-HT_1A_ receptor stimulation

5-HT_1A_ agonists and antagonists modulate 5-HT neurotransmission and have been shown to directly alter spatial learning performance. Typically, antagonists have been found to impair spatial memory, whereas agonists are found to ameliorate the antagonist-induced spatial deficits, or allowed normal performance. For example, in a study by Micheau and Van Marrewijk (1999), intra-peritoneal administration of the 5-HT_1A_ receptor agonist 8-hydroxy-2-(di-*n*-propylamino) tetraline (8-OH-DPAT) *improved* acquisition of a spatial discrimination task in an 8-arm radial maze. An intra-septal infusion of 8-OH-DPAT produced the same pattern of findings, although the improvement was less pronounced. Bertrand et al. (2000) showed contradictory findings, however, reporting that intra-septal infusion of 8-OH-DPAT *impaired* spatial learning. Administration of 8-OH-DPAT into the rat *dorsal raphe* had no effect on Delayed Non-Matching to Position (spatial working memory) task performance at any dose. In comparison, administration of the same compound into the *median raphe* improved performance accuracy. When 8-OH-DPAT was administered into the dorsal hippocampus, however, it produced a small impairment in performance (Warburton et al., 1997). 8-OH-DPAT also impaired performance on a water maze task (Carli et al., 1995) and on the eight-arm radial maze (Egashira et al., 2006). These findings demonstrate different effect for pre- and post-synaptic 5-HT_1A_ receptor stimulation on spatial learning and memory tasks.

Additional evidence for the role of 5-HT_1A_ receptor agonists in spatial memory comes from animal models of traumatic brain injury (TBI). In this model, animals are subjected to controlled cortical lesion to mimic TBI, and then memory is examined at different time points following injury and after administration of 5-HT_1A_ agonists. These studies showed that TBI-induced spatial memory deficits are attenuated by treatment with the 5-HT_1A_ receptor agonist buspirone (Olsen et al., 2012) and 8-OH-DPAT (Cheng et al., 2008). Furthermore, a combined therapeutic regimen of buspirone and environmental enrichment was found to be more effective than either alone in enhancing spatial learning in brain injured pediatric rats (Monaco et al., 2014).

### Imaging serotonergic neurotransmission

Because the 5-HT_1A_ receptor plays an important role in a range of physiological processes and in the pathophysiology of a variety of psychiatric and neurodegenerative disorders, synthesis of 5-HT_1A_ receptor agents has been carried out primarily for their therapeutic potential. In recent years, more than 20 compounds have been labeled with carbon-11, fluorine-18, or iodine-123 for imaging and quantification of the 5-HT_1A_ receptor with PET and SPECT (for review see Passchier and Van Waarde, 2001). The most successful radioligands thus far are [*carbonyl*-^11^C] WAY-100635 (WAY), [*carbonyl*-^11^C]*desmethyl*-WAY 100635 (DWAY), 2′-methoxyphenyl-(*N*-2′-pyridinyl)-*p*-[^18^F]fluoro-benzamidoethylpiperazine ([^18^F]MPPF), and [^11^C]robalzotan (NAD-299) (Passchier and Van Waarde, 2001). To the best of our knowledge, the only study that examined 5-HT_1A_ receptor density and spatial learning and memory (i.e., object-location, navigation, and floor plan drawing) in humans using the PET ligand [^18^F]MPPF was recently published by our group (Glikmann-Johnston et al., 2015). In this study, healthy participants performed spatial virtual environment tasks during PET scanning. We found an association between hippocampal asymmetry in [^18^F]MPPF binding and performance on the object-location task. A lower binding potential in the right vs. the left hippocampus was related to better memory performance. This finding indicates that reduced right vs. left hippocampal 5-HT_1A_ receptor availability enhances object-place associative memory. Although not within the scope of this review, it is important to note that Theodore et al. (2012) used similar experimental methodology in verbal memory using the 18FCWAY PET ligand. In their study, reduced left hippocampal 5-HT_1A_ receptor binding in temporal lobe epilepsy (TLE) patients was related to delayed auditory verbal memory impairment, independent of the side of the epileptic focus. More cognitive serotonergic imaging studies are needed to build up the evidence for the role of 5-HT_1A_ receptor in fundamental components of human spatial memory.

### Genetic variance in 5-HT_1A_ receptor availability

Congenital differences in 5-HT_1A_ receptor availability were found to be related to spatial memory, specifically length variations in the serotonin-transporter-gene-linked polymorphic region (5-HTTLPR). 5-HTTLPR is a 44-base pair insertion/deletion functional polymorphism in the promotor region of the serotonin transporter (5-HTT) gene (Lesch et al., 1996). This polymorphism produces two common alleles designated long (L) and short (S), and was found to affect 5-HT_1A_ receptor availability (David et al., 2005). Human (Roiser et al., 2006, 2007) and primate (Jedema et al., 2010) carriers of S allele demonstrated superior performance compared to L carriers on a variety of cognitive tasks, including hippocampal-dependent visual memory tasks (a computerized version of the Block Design subtest of the Wechsler Adult Intelligence Test and the CANTAB Pattern Recognition Memory and Delayed Match to Sample).

### Manipulations of 5-HT levels

Pharmacological alterations of 5-HT concentrations, by altering either 5-HT release or reuptake, have been shown to influence spatial memory. Overall, increased extracellular 5-HT concentrations maintain or improve memory performance, and reduced levels of the neurotransmitter impair spatial memory. Changes in 5-HT release are thought to indirectly stimulate post-synaptic 5-HT_1A_ receptors, which reside on areas important to spatial learning and memory, thereby affecting memory function (Lesch et al., 1996; Kuypers and Ramaekers, 2005). Support for this hypothesis is found in a study by du Jardin et al. (2014) with the use of parachlorophenylalanine (PCPA). This compound inhibits tryptophan hydroxylase, and thus reduces 5-HT synthesis. In their study, PCPA induced 5-HT depletion in rats and caused memory deficits on object recognition and Y-maze spontaneous alternation tests. The selective 5-HT_1A_ receptor agonist flesinoxan significantly occupied 5-HT_1A_ receptors and restored PCPA-induced memory deficits in both tests. Although other agents had similar effects on spatial memory function (e.g., ***3,4-methylenedioxymethamphetamine/MDMA***: Fox et al., 2000; Skelton et al., 2006; Vorhees et al., 2007; Fisk et al., 2011; ***D-fenfluramine***: Morford et al., 2002; ***methamphetamine***: Vorhees et al., 1994, 2000, 2008; Schröder et al., 2003), studies to date did not involve the 5-HT_1A_ receptor directly. Even though the 5-HT_1A_ receptor is the most abundant in the hippocampus, it is not possible to exclude other receptor subtypes that 5-HT stimulate in this area (5-HT_2A_, 5-HT_6_, and 5-HT_7_), and that may have an effect on spatial memory.

## Conclusion

The findings reviewed here provide converging evidence in support of the hypothesis that 5-HT, mediated by the 5-HT_1A_ receptor, plays a key role in hippocampal-dependent spatial memory in animals and humans. Strong evidence comes from knockout mouse models. These studies have shown that 5-HT_1A_ receptor knockouts are specifically associated with deficits in performance on spatial memory tasks. A variety of agonists and antagonists active at the 5-HT_1A_ receptor modulate 5-HT neurotransmission and induce a change in spatial learning. Blockade of the 5-HT_1A_ receptor impairs spatial memory, while receptor activation ameliorates antagonist-induced spatial memory deficits. Another line of evidence emerges from studies that vary neurotransmitter levels pharmacologically. Typically, increased 5-HT extracellular concentrations maintain or improve memory performance, and reduction in neurotransmitter levels impairs spatial memory.

Recent advances in human neurotransmitter research methods allow for more direct quantification of 5-HT_1A_ receptor availability during spatial learning and memory. Initial results from neuroimaging studies with the use of neurotransmitter PET indicate the contribution of endogenous serotonin release or 5-HT_1A_ receptor density to spatial memory, particularly to the ability to recall the location of objects in the environment (Glikmann-Johnston et al., 2015). The mapping of the human genome provides further evidence at the individual person level for the association between 5-HT_1A_ receptor density and spatial memory.

Theories of hippocampal involvement in spatial memory include: (a) the cognitive map theory of O'Keefe and Nadel (1978); (b) the theory proposed by Olton and colleagues (Olton et al., 1979; Olton and Paras, 1979), in which the hippocampus is crucial for working memory; and, (c) the theory that attributes a binding mechanism to the hippocampus to form spatial memories such as object location (for example, Chalfonte et al., 1996; Eichenbaum et al., 1996). The evidence reviewed in this paper involving 5-HT, particularly the 1A receptor subtype, and spatial memory is further supported by the well-established notion of the involvement of the hippocampus in spatial memory function.

A substantial number of studies have examined the role of 5-HT in spatial learning and memory and have demonstrated, particularly in animals, a strong relation between 5-HT and spatial memory. Yet several significant questions remain. We suggest that additional research is needed to clarify the relationship between 5-HT_1A_ receptor modulation and specific aspects of spatial memory, including object location and spatial frames of reference, allocentric vs. egocentric representations, and navigation and episodic memory within a topographical framework (Burgess et al., 2002; Burgess, 2008). Also, research is needed into how the serotonergic system interacts with other major neurotransmitter systems, including the acetylcholineric system, to modulate spatial memory.

For patients with damage to the temporal lobes due to progressive pathology such as Alzheimer's disease, impairments of spatial memory are often the first symptoms reported. The idea that hippocampal 5-HT_1A_ receptor plays a key role in spatial learning and memory may be informative for early intervention strategies, and for improving patient outcomes in diseases affecting the temporal lobes.

## Author contributions

YG-J, MS, DR, and JS wrote the article, reviewed the article, and approved the final version for publication.

### Conflict of interest statement

The authors declare that the research was conducted in the absence of any commercial or financial relationships that could be construed as a potential conflict of interest.
